# Patients’ and physicians’ satisfaction with a pharmacist managed anticoagulation program in a family medicine clinic

**DOI:** 10.1186/s13104-015-1187-8

**Published:** 2015-06-09

**Authors:** Lisa Bishop, Stephanie Young, Laurie Twells, Carla Dillon, John Hawboldt

**Affiliations:** School of Pharmacy, Memorial University, 300 Prince Philip Drive, St. John’s, NL A1B 3V6 Canada; Faculty of Medicine, Memorial University, 300 Prince Philip Drive, St. John’s, NL A1B 3V6 Canada

**Keywords:** Questionnaires, Ambulatory care, Family practice, Primary health care, Pharmacists, Warfarin, Anticoagulants, Medication therapy management

## Abstract

**Background:**

A pharmacist managed anticoagulation service was initiated in a multi-physician family medicine clinic in December 2006. In order to determine the patient and physician satisfaction with the service, a study was designed to describe the patients’ satisfaction with the warfarin education and management they received from the pharmacist, and to describe the physicians’ satisfaction with the level of care provided by the pharmacist for patients taking warfarin. A self-administered survey was completed by both eligible patients receiving warfarin and physicians prescribing warfarin between December 2006 and May 2008. The patient survey collected information on patient demographics, satisfaction with warfarin education and daily warfarin management. The physician survey collected data about the satisfaction with patient education and daily anticoagulation management by the pharmacist.

**Results:**

Seventy-six of 94 (81%) patients completed the survey. Fifty-nine percent were male with a mean age of 65 years (range 24–90). Ninety-six percent agreed/strongly agreed the pharmacist did a good job teaching the importance of warfarin adherence, the necessity of INR testing and the risks of bleeding. Eighty-five percent agreed/strongly agreed the risk of blood clots was well explained, 79% felt the pharmacist did a good job teaching about dietary considerations and 77% agreed/strongly agreed the pharmacist explained when to see a doctor. All patients felt the pharmacist gave clear instructions on warfarin dosing and INR testing. Four of nine physicians (44%) completed the survey. All agreed/strongly agreed the pharmacist was competent in the care provided, were confident in the care their patients received, would like the pharmacist to continue the service, and would recommend this program to other clinics.

**Conclusions:**

Patients and family physicians were satisfied with the pharmacist managed anticoagulation program and recommended continuation of the program. These results support the role of the pharmacist in the management of anticoagulation in a multi-physician family medicine clinic.

## Background

Warfarin therapy is well established and has been the main oral anticoagulant used for the prevention and treatment of thromboembolism. Patients taking warfarin require close blood monitoring to maintain their international normalized ratio (INR) within therapeutic range to ensure effectiveness and to minimize the risk of bleeding. This is challenging in clinical practice due to inter-patient variability and the susceptibility of warfarin to pharmacokinetic changes due to concurrent medications, diet, and various disease states [1]. The Institute for Safe Medication Practices (ISMP) maintains a list of high alert medications, which includes warfarin [2]. ISMP noted that when used or omitted in error, anticoagulants, such as warfarin, can cause life threatening bleeding or thrombosis.

The American College of Chest Physicians (ACCP) guidelines recommends that “health-care providers who manage oral anticoagulation therapy should do so in a systematic and coordinated fashion, incorporating patient education, systematic INR testing, tracking, follow-up, and good patient communication of results and dosing decisions” [1]. Evidence indicates that a coordinated pharmacist managed anticoagulation program is associated with less adverse events and hospitalization admissions as compared to usual care [3, 4]. A recent meta-analysis indictated that pharmacist-participated warfarin management therapy significantly reduced total bleeding, with a non-significant trend towards decreases in other warfarin-related complications [5]. Despite this evidence, many anticoagulated patients do not receive care from such services. Optimum delivery of anticoagulation therapy should be a priority of all clinicians involved in the care of patients receiving anticoagulation therapy. Required components for optimized delivery and evaluation of these specialized services exist [6].

Several target specific oral anticoagulants (TSOAC) are available in Canada which have more predictable pharmacologic and pharmacokinetic properties than warfarin, thus eliminating the need for frequent INR monitoring [7–9]. Due to the ease of administration and reduced monitoring of these agents, they may replace the use of warfarin for many indications [10, 11]. Known contraindications, emerging reports of serious adverse events, and lack of anticoagulation reversibility results in situations where warfarin remains the drug of choice [10, 12]. The principles of patient selection, provider education and training, interruption of treatment for invasive procedures, and careful follow-up will remain, resulting in a reliance on specialized anticoagulation programs to effectively manage anticoagulation.

Generally, patient and physician satisfaction with anticoagulation programs is reported as high [13–17]. Willey et al. [13] evaluated satisfaction surveys mailed to patients and referring physicians of a pharmacist-managed outpatient anticoagulation clinic. Overall, both patients and physicians were very satisfied with the clinic and physicians indicated the care provided by the clinic decreased their workload. Significant differences favoring the presence of anticoagulation clinics have also been noted in several studies [14, 15, 17]. In these studies, patient satisfaction was measured in anticoagulation clinics and then compared to physician-managed care. Finally, one study found in the literature examined pharmacist anticoagulation management integrated into a private physician office setting [16]. All patients agreed or strongly agreed that they were satisfied with the overall service provided to them by the pharmacist. There is limited information on patient and physician satisfaction with anticoagulation programs in private physician office settings.

In Newfoundland and Labrador (NL), Canada, outpatients on warfarin are generally managed by their family physician. In 2006, a pharmacist-run anticoagulation service was initiated at a family medicine clinic in NL. The pharmacist obtained the INR result from the local laboratory, contacted patients primarily via telephone, and used an electronic medical record to document the encounters and communicate with the physicians. An evaluation of the anticoagulation service demonstrated that the pharmacist provided service achieved significantly better INR control as compared to usual care [18]. The specific objectives of the present study were to describe patients’ satisfaction with the warfarin education and management they received from the pharmacist, and to describe the physicians’ satisfaction with the level of care provided by the pharmacist for their patients taking warfarin. Information from this research will be used to support the role of the pharmacist in the management of anticoagulation in a family medicine clinic.

## Methods

### Selection of study participants

The study was conducted in August 2008, with sampling of all patients who received anticoagulation management by the pharmacist at the family practice clinic and all physicians who prescribed warfarin between December 7, 2006 and May 7, 2008. All patients enrolled in the program and who had at least one INR blood test that was managed by the pharmacist between this time period were included in the study. Patients were excluded if they were deceased or if a current address could not be located.

### Setting

The family practice clinic is located in St. John’s, NL, a capital city with a population of approximately 200,600 [19]. In December 2006, the primary healthcare pharmacist at the family practice clinic initiated an anticoagulation program, which was the first of its kind in NL. The program was primarily a telephone-based service to educate, counsel, and instruct patients on the use of warfarin, which was overseen by the family physicians. Written patient educational material was provided to all the patients which summarized the education provided by the pharmacist regarding their warfarin therapy. This material was developed by the pharmacists. A physician-approved protocol was used by the pharmacist for the education and day-to-day management of patients taking warfarin. Additional details about the service have been described previously [18].

### Survey method

All eligible patients and physicians were mailed an anonymous survey printed on bright, colored paper, with an accompanying cover letter and self-addressed, return stamped envelope. A follow-up reminder letter and survey was mailed 2 weeks after the first survey to all eligible participants. Completion of the survey was considered consent to participate.

### Survey design

Literature searches of PubMed, EMBASE, IPA and ProQuest Dissertations and Theses were conducted to identify existing patient and physician surveys on satisfaction with anticoagulation programs. If needed, study authors were contacted. Existing surveys were taken into consideration when developing the surveys used in the present study, along with articles on how to design questionnaires [13, 14, 16, 20–26]. The surveys were designed with input from all research team members.

The patient survey consisted of 14 statements which gathered information about the warfarin education provided by the pharmacist, as well as opinions around the daily management of warfarin, including instructions regarding the dose of warfarin, when to get an INR test, if calls from the pharmacist were returned, if the pharmacist could answer questions, if the pharmacist was courteous and respectful, if the patients were aware of the pharmacists collaboration with other health professionals, and overall service satisfaction. Demographic information collected included sex, year of birth, highest level of education, duration of warfarin use, and how the participant’s warfarin was managed prior to the pharmacist.

The physician survey consisted of eleven statements, which gathered information concerning level of satisfaction with patient education provided by the pharmacist as well as the daily management of their warfarin patients. Questions included whether they felt the pharmacist was competent, if they now felt more comfortable managing patients on warfarin, if they were contacted if issues arose, if they were kept up-to-date on therapy changes, if their time on warfarin issues had decreased, if adverse events had decreased, and their overall opinion of the service.

Opinions for both surveys were captured via a 5-point Likert scales ranging from strongly agree to strongly disagree. For these questions, responses were collapsed to three categories of “agree” (included strongly agree and agree), “uncertain”, and “disagree” (included strongly disagree and disagree), and reported as percentage of agreement or disagreement for each statement. Open responses were also captured.

The questions as they appeared on the surveys are included in the results tables (Tables 1, 2, 3).

**Table 1:** Patient satisfaction with warfarin education from pharmacist (n = 76)

Statement	n (%)			
	Agree or strongly agree	Uncertain	Disagree or strongly degree	Missing
The clinic pharmacist did a good job teaching me about:				
Why I should take my warfarin everyday	69 (96)	3 (4)	0	4
Why I need to have my INR blood test done	72 (97)	2 (3)	0	2
The risks of bleeding which may occur with warfarin	70 (95)	3 (4)	1 (1)	2
The risk of a blood clot if my INR blood test is too low	62 (85)	9 (12)	2 (3)	3
When I need to see a doctor or go to the emergency room	56 (77)	14 (19)	3 (4)	3
What foods may affect warfarin	57 (79)	12 (17)	3 (4)	4

**Table 2:** Patient satisfaction with warfarin management from pharmacist (n = 76)

Statement	n (%)			
	Agree or strongly agree	Uncertain	Disagree or strongly disagree?	Missing
The clinic pharmacist gave me clear instructions about what dose of warfarin to take	75 (100)	0	0	1
The clinic pharmacist gave me clear instructions about when to get my INR blood test done	75 (100)	0	0	1
The clinic pharmacist returned my calls in a timely fashion	73 (99)	1 (1)	0	2
The clinic pharmacist treated me with courtesy and respect	75 (100)	0	0	1
I trust the clinic pharmacist to answer my questions about warfarin	75 (100)	0	0	1
I am satisfied with the service provided by the clinic pharmacist in managing my warfarin	75 (100)	0	0	1
The clinic pharmacist worked with other health care professionals to help manage my warfarin	53 (77)	15 (22)	1 (1)	7
Other patients taking warfarin would benefit from this type of service	74 (100)	0	0	2

**Table 3:** Physician satisfaction survey results (n = 4)

Statement	n (%)		
	Agree or strongly agree	Neutral	Disagree or strongly disagree
The clinic pharmacist is competent in the management of warfarin	4 (100)	0	0
I am more confident in initiating or managing new patients on warfarin when the clinic pharmacist is involved	4 (100)	0	0
I am contacted appropriately by the clinic pharmacist when issues arise with warfarin management for my patients	4 (100)	0	0
I am kept up-to-date on changes in warfarin therapy for my patients in a timely manner	4 (100)	0	0
Warfarin management by the clinic pharmacist has decreased the number of questions I receive from patients about warfarin	3 (75)	1 (25)	0
Warfarin management by the clinic pharmacist has decreased the amount of time I spend on anticoagulation issues for my patients	3 (75)	1 (25)	0
My patients have a better understanding of warfarin therapy since they have been managed by the clinic pharmacist	4 (100)	0	0
The clinic pharmacist managed warfarin program has improved continuity of care for my patients	4 (100)	0	0
Close monitoring of warfarin by the clinic pharmacist has decreased the likelihood of adverse events in my patients	4 (100)	0	0
I would like to continue having the clinic pharmacist manage warfarin in my patients	4 (100)	0	0
I would recommend to my colleagues in other clinics to initiate a pharmacist-managed warfarin services	4 (100)	0	0

### Statistical analysis

A sample size calculation was not required given that all eligible the patients and physicians were surveyed. Data was entered into SPSS statistical software for Windows (version 16.0) spreadsheet for analysis. Descriptive statistics were used for all questions.

### Ethics approval

The Human Investigations Committee at Memorial University approved the research protocol.

## Results

### Patient survey

Of the 112 patients managed by the pharmacist, 94 met the eligibility criteria after exclusion of 18 patients (six were deceased and 12 could not be located). All eligible patients were mailed surveys and 76 were returned, providing a response rate of 81%. The demographics of the patients are reported in Table 4. The majority of patients were male with a mean age of 65. At the time of the survey, most patients (47%) reported taking warfarin for 1–5 years, 28% reported more than 5 years of use and 24% less than 1 year of therapy. The majority (81%) of patients reported being managed by their family physician prior to the pharmacist-managed program, while 12% were only managed by the pharmacist, and 7% indicated “not sure or not applicable”.

**Table 4:** Patient baseline characteristics (n = 76)

Respondent characteristics	Result n*	%
Sex		
Male	42	59
Female	29	41
Age years, mean (SD)	65 (16)	
Min, max in years	24–90	
Highest level education		
Post-secondary	47	66
High School	15	21
Grade School	9	13

* Not all respondents replied to each question.

Almost all patients were satisfied with the warfarin education provided by the pharmacist. At least 95% of patients agreed/strongly agreed that the pharmacist completed a satisfactory job teaching the importance of warfarin adherence, why INR tests were necessary, and the risks of bleeding. Approximately 75% agreed/strongly agreed that the risk of a blood clot was explained, that dietary considerations for warfarin therapy were described, and that they were told when to see a doctor. Table 1 outlines the breakdown of responses regarding patient satisfaction with the warfarin education.

All patients reported being satisfied with the serviced provided by the pharmacist and reported as “trusting” the pharmacist (Table 2). All patients also agreed/strongly agreed that the pharmacist gave clear instructions on the dose of warfarin and when to get their INR test repeated. Over 75% agreed/strongly agreed that the pharmacist worked with other health care providers in the management of their warfarin.

Forty-six patients provided additional written comments, which were all positive. Examples of comments included: “…the pharmacist warfarin program is a great addition and hope it will continue”; “It is a useful service. Much better than just dealing with your physician for INR testing and results”; “I was getting comprehensive care”; “More doctors’ offices should be using it!”; “…made everything easy and consistent”; “It’s reliable, dependable and more thorough.”

### Physician survey

Of the nine physicians who were mailed a survey, four (44%) completed the survey. All the physicians indicated they were happy with the pharmacist-managed program and agreed/strongly agreed that other clinics should initiate a similar program. Three-quarters of the physicians agreed/strongly agreed that the program decreased the number of questions from patients about warfarin and decreased the amount of time they spent on anticoagulation issues. Details of the physician satisfaction results are listed in Table 3.

Additional written comments were provided by the physicians who responded to the survey. The comments were all positive, including such things as “great service”; “…more in depth care and communication with the patient”; “Patients love the ability they have to contact pharmacist re: the warfarin and any questions they have regarding their medications…”; “… always stay within their scope and refer to doctors appropriately and keep us informed”.

## Discussion

The results of this study indicate that the patients who received care from the pharmacist-managed anticoagulation program were satisfied with the service provided and felt that this program should be expanded to other family medicine clinics. Most patients were also satisfied with the education they received, which included information about why they needed to take warfarin, the risks of bleeding, and potential thromboembolism. The physicians were also satisfied with the program, with all physicians feeling confident in the care provided to the patients and indicating that other family physician offices could benefit from such a service.

The overall satisfaction of our program from patients who received care was 100%, which is similar to other studies. Overall patient satisfaction with anticoagulation services has been cited in the literature as ranging from 95 to 100% [13–17]. Garwood et al. [14] considered whether transitioning patients from a pharmacist-managed anticoagulation clinic to a physician-managed care alters the quality of care. Statistically significant differences favoring pharmacist-managed anticoagulation were noted in all areas of clinical care. These results suggested that the patients believed the pharmacist had more expertise in managing anticoagulation control when compared with their physician. Although our study did not look at the transition from pharmacist management back to physician management, all patients indicated that they trusted the pharmacist to answer their questions, and over three quarters were satisfied with the anticoagulation education they received.

Additional studies have demonstrated patient and physician confidence in pharmacist managed anticoagulation. In a community-pharmacy led anticoagulation service, attitudes towards a pharmacist model of management showed that most patients (79%) and physicians (89%) were confident in the pharmacist’s ability to manage therapy [26]. In an outpatient pharmacist-managed anticoagulation clinic that was associated with a local hospital, both patient and physicians demonstrated a high level of satisfaction with the service [27].

The setting of our study was a private family physician office, which was similar to a study by Ernst et al. [16]. In this study they examined the anticoagulation management that occurred in a private physician office setting. Results indicated that all patients agreed that they were satisfied with the overall service provided to them by the pharmacist, felt the pharmacist was courteous, felt they increased their knowledge of warfarin, and would recommend this service to other patients. The physicians also reported satisfaction with the pharmacist-run service, demonstrating that the clinic is convenient, provides better continuity of care, results in fewer questions, and would prefer to have patients handled through the anticoagulation clinic. These findings are consistent with our study, which emphasized the benefit of pharmacist-managed anticoagulation programs in a private family office setting.

An evaluation of our pharmacist-managed anticoagulation service demonstrated a significantly better INR control for the pharmacist-managed care as compared to usual care by the physicians, with the time in therapeutic range 73 vs. 65%, respectively (p < 0.0001) [18]. This current study adds to the evidence supporting the benefit of the service by showing that patients and physicians are satisfied with a telephone based program in a private family physician office. Since pharmacists who work directly in a family practice setting is limited in NL, the results of this study support exploring the extension of this model to other family medicine practice sites. The impact of the TSOAC on the current anticoagulation programs in family medicine would be an interesting area of future research.

There are several strengths to our study. The response rate from both the patients (81%) and physicians (44%) was comparable or better than other similar studies [13–17, 26, 27]. We also increased our response rate to the survey by mailing out a second survey to all potential participants 2 weeks after the original survey as a reminder to complete the survey. The survey was anonymous and did not collect any identifying data so the patients and physicians should have felt comfortable responding to the survey.

There were some limitations to our study. This was a retrospective cross-sectional survey so some patients may have had difficulty remembering their encounters with the pharmacist if they used the service for only a short time period or had not used the service recently. In addition, many of the patients were on warfarin before the anticoagulation service was established while others initiated warfarin using the pharmacist-run service. This may have influenced the responses of the patients, in particular the questions about the education they received from the pharmacist. Our study did not have a comparison with usual physician managed care. Since the pharmacist took over the management of warfarin in 2006, we felt that there would be inaccuracies in patients recalling their experiences prior to this time. As well, we did not capture the patient’s perceptions of their INR and warfarin control since being managed by the pharmacist. We did not feel that this was necessary as the patient’s INR control was systematically collected over the same time period and was shown to be better than usual care [18]. Although the survey tool was designed based on previously published survey tools used in the literature, the survey tools were not validated for evaluating an anticoagulation service. Some factors that may limit the generalizability of the results, as it was conducted at a single site and there were only four physician responses to the survey. This study was also based on a service from 2006 to 2008; however, this data is still applicable as warfarin remains a commonly used anticoagulant in practice.

## Conclusions

Patients and family physicians were satisfied with the pharmacist-managed anticoagulation program in a multi-physician family medicine clinic and recommended continuation of the program. The service is highly valued by both patients and family physicians and can support the role of the pharmacist in the management of anticoagulation in a multi-physician family medicine clinic.
